# Trickle-down effects of temporal leadership: The roles of leadership perspective and identification with leader

**DOI:** 10.3389/fpsyg.2022.1013416

**Published:** 2022-09-16

**Authors:** Yingying Zhang, Zhonghui Hu, Siyu Tian, Chunyang Zhou, Yi Ding

**Affiliations:** ^1^College of Business, Shanghai University of Finance and Economics, Shanghai, China; ^2^Business School, University of Shanghai for Science and Technology, Shanghai, China

**Keywords:** temporal leadership, temporal leadership perspective, trickle-down effects, social learning theory, identification with a leader

## Abstract

Based on social learning theory and the trickle-down effects, in which behavioral patterns cascade from one management level to the next (also known as the falling domino effect), we attempt to answer whether upper-level managers’ temporal leadership can be transferred to lower-level managers to form their temporal leadership, and what the mediating mechanisms and boundary conditions for this occurrence are. By analyzing the data from 234 middle-level managers and 686 junior managers/employees, we found that top managers’ temporal leadership was positively associated with middle-level managers’ temporal leadership through the mediating role of middle-level managers’ temporal leadership perspective and that the relationship was moderated by middle-level managers’ identification with the top manager. Identification with the top manager, in particular, strengthens both the top manager’s positive effect on middle-level managers’ temporal leadership and the top manager’s temporal leadership’s mediating role in this relationship through their temporal leadership perspective. The theoretical and managerial implications of these findings are investigated.

## Introduction

Temporal leadership refers to controlling the temporal components of team activities, such as temporal synchronization and the allocation of temporal resources (Mohammed and Nadkarni, 2011). There has been a significant increase in research on this topic over the last decade (Chen and Nadkarni, 2017). Temporal leadership provides a micro foundation for researchers to understand the relational temporal resource management process within the organization, and it assists organizations in dealing with temporal challenges in this VUCA world (e.g., dramatic changes in the business environment, technological updating, and/or rapid shifts in customer preferences; Shipp and Fried, 2014; Chen and Nadkarni, 2017). Considering temporal leadership is effective in promoting employees’ performance, team performance, and even organizational performance (Gevers et al., 2009; Op't Hoog, 2009; Mohammed et al., 2015; Mudannayake et al., 2016; Yuan and Lo, 2018), it is not surprising that organizations are increasingly eager to know what motivates leaders to exhibit temporal leader behaviors and how to develop managers’ ability to navigate temporal resources (Waller et al., 2001; Gevers et al., 2006).

Despite the fruitful findings on nomological networks of temporal leadership (e.g., Hubens, 2011; Mohammed and Alipour, 2014; Mudannayake et al., 2016; Chen and Nadkarni, 2017; Yuan and Lo, 2018; Xiao et al., 2020), we still know little about how temporal leadership spreads across hierarchical levels. We argue that the limited focus is unfortunate for two reasons. First, while the dispositional perspective may explain some variance in temporal leadership, the occurrence of temporal leadership may not be attributable to individual traits alone (e.g., Kirkpatick and Locke, 1991; Chen and Nadkarni, 2017). More antecedents should be explored to explain how managers perform temporary leadership. Secondly, the research on the outcomes of temporal leadership focuses on employees’ work behaviors and performance, without distinguishing the hierarchy of leaders, which leaves an unanswered question about why middle-level or line managers exhibit temporal leadership and how temporal leadership cascades from an upper level to a lower level. According to research on the trickle-down effects of behaviors, managers tend to mimic their upper-level supervisors’ behaviors; how upper-level managers treat their middle-level subordinates influences how middle-level managers treat employees (Mayer et al., 2009).

We turn to social learning theory to shed light on the antecedents of middle-level managers’ temporal leadership from a trickle-down approach (e.g., Bandura, 1977, 1986) and focus on the role modeling process concerning temporal leadership. Leadership research, includes transformational leadership (Bass et al., 1987), ethical leadership (Mayer et al., 2009), abusive leadership (Aryee et al., 2007; Liu et al., 2012; Mawritz et al., 2012), and family-supportive leadership (Kwan, 2014), suggests that followers imitate the leadership behaviors they observe from their leaders because the leaders are usually considered as role models. Individuals learn behaviors by observing environmental cues, and they typically prefer to learn from role models to avoid trial-and-error costs, according to social learning theory (Lian et al., 2012). Although role models can be ubiquitous, individuals are more likely to consider individuals with higher status as role models and then imitate their behavior (Bandura, 1986). The top managers are usually regarded as the symbols of a high-status model within the organizations and the lower-level managers tend to mimic their behavior (Liu et al., 2013). Based on social learning theory, we argue that top managers’ temporal leadership should drive the temporal leadership of middle-level managers (Bandura, 1977).

To untangle the complexities in the trickle-down processes of temporal leadership, we further examine the mechanisms underlying the relationship between top managers’ temporal leadership and middle-level managers’ temporal leadership. We propose that upper-level managers influence middle-level managers through the mediating role of the temporal leadership perspective, which we define as the extent to which the leader explains his or her role as supervisor in scheduling deadlines, synchronizing behaviors, and allocating temporal resources. It is known from previous management literature that managers’ definition of their job responsibilities is influenced by their exposure to a variety of social and behavioral cues (Salancik and Pfeffer, 1978). The differences in job responsibilities generated by these cues are related to the differences in the “schemas” of leaders’ subjective perceptions of their job responsibilities (Morrison, 1994). The foundation of social learning theory is the concept of “schemas.” They influence the types of social information that individuals pay attention to, categorize, interpret, store, and recall from memory (Baldwin, 1992). Explains how individuals learn from their surroundings by mentally organizing and representing information about themselves and others (Bandura, 1986). While personal perspective is an important schema, individuals express learned behaviors through perspective modification. Individuals will find appropriate cognitive scripts (i.e., perspectives) when they observe certain “correct” behaviors of role models, which will then guide their subsequent behaviors in social interactions (Gioia and Poole, 1984; Freeman and Martin, 2004).

The distinction between temporal leadership and temporal leadership perspective is that temporal leadership are process improvement behaviors (Mohammed and Alipour, 2014), a series of behaviors that leaders aim to help subordinates allocate temporal resources, whereas temporal leadership perspective is a belief in the behaviors. The willingness of the leader supervisor to do what needs to be done to support and assist subordinates in allocating temporal resources. Although beliefs frequently translate into behavior, research has shown that the impact of beliefs on behavior is determined by three factors: Individuals’ judgments of their ability to perform the action, their perception of the rewarding or punishing consequences of such behavior in previous events, and the likelihood of similar or different consequences if such behavior is demonstrated (Bandura, 1986). Disincentive effects on performance behavior occur when individuals are unable to perform the act, when they observe the punishment elicited by the act, and when they predict that if they engage in similar behavior, they will be punished rather than rewarded. As a result, temporal leadership perspective can guide leaders to recall their supervisors’ temporal leadership, interpret these temporal leadership as behaviors that are conducive to subordinates’ use of temporal resources and resolution of temporal conflicts, and thus generate the willingness to help subordinates allocate and manage temporal resources and insist on providing their own temporal leadership. In this regard, we propose that temporal leadership cascades across hierarchies by activating lower-level managers’ perspective systems, which a supervisor should do to manage subordinates’ temporal resources.

However, not all employees consider their managers to be role models. Based on social learning theory, we claim that middle-level managers’ identification with their top manager moderates the temporal leadership trickle-down process. Identification with a supervisor refers to the extent to which individuals incorporate the supervisor into their relational self (Kark et al., 2003; Wang and Rode, 2010). Research has found that the role modeling process can be facilitated when a person’s experience with a supervisor becomes self-defining or self-referential (Kark et al., 2003), which means that the self is associated with important others and each association contains a self-other relationship (Andersen and Chen, 2002). They are willing to imitate role models based on their observations (Rosenhan and White, 1967; Lord and Brown, 2004). Thus, we argue that temporal leadership is more likely to be transmitted when middle-level managers have a higher degree of identification with their leaders. By identifying the mechanism and boundary conditions of the temporal leadership trickle-down process, we can explain how this style of leadership can transmit through a level of hierarchy and thus facilitate the management process of temporal resources.

Our contributions to the current literature are mainly threefold. First, we contribute to the literature on temporal leadership by revealing temporal leadership’s trickle-down effects. To the best of our knowledge, this is the first empirical study to analyze why lower-level (e.g., middle-level) managers demonstrate temporal leadership in contrast to upper-level managers (Chen and Nadkarni, 2017). By addressing this research gap, we are willing to provide a more comprehensive picture of temporal leadership antecedents and outcomes, and also provide practical insights toward fostering temporal leadership. Second, through social learning theory, we highlight the mediating role of the temporal leadership perspective and contribute to the literature on temporal leadership. By incorporating the concept of temporal leadership perspective into the trickle-down process of temporal leadership, we respond to the call of temporal leadership scholars for more studies on temporal cognition (e.g., Mohammed and Alipour, 2014; Santos et al., 2016). Third, we extend our understanding of temporal leadership by revealing the boundary condition of temporal leadership’s trickle-down effects. In our proposed model, we emphasize the significance of the identification process (i.e., middle managers’ identification with their supervisor; Lord and Brown, 2004) based on social learning theory.

## Theory and hypotheses

### Top managers’ temporal leadership and middle-level managers’ temporal leadership

Leaders play an important role in impacting members’ attitudes and behaviors in the organization (Bass et al., 1987). Indeed, the leadership styles of higher-level managers can influence that of lower-level managers through their effects on cognition and behavior (Bass et al., 1987; Cheng et al., 2019; Byun et al., 2020). For example, Cheng et al. (2019) found that high-level leader responsible leadership impacted low-level leader responsible leadership. In addition, Bass et al. (1987) demonstrated that transformational leadership could appear by chance at different hierarchical levels and then immediately transfer to other levels. In line with this stream of research, we propose that temporal leadership, defined as leaders’ behaviors in controlling the temporal components of team activities, such as temporal synchronization and the allocation of temporal resources (Mohammed and Nadkarni, 2011), can transmit across hierarchical levels.

If top managers often display temporal leader behaviors (e.g., asking followers to meet deadlines, keeping the pace of the work process, and reducing time conflicts during the work process), middle-level managers will learn behaviors that emphasize the rational use of time resources are encouraged in organizations. When middle-level leaders consider making full use of time resources as norms of their organizations, they tend to adopt corresponding behavioral patterns as their benchmarks and even internalize these norms as their own management style. In addition, temporal leadership is a positive behavior that can benefit an organization in a variety of ways, including accelerating internal organizational functioning, increasing employee productivity, job performance, and enhancing subordinate creativity, as well as reducing time conflicts in work tasks (Mohammed and Alipour, 2014; Chen and Nadkarni, 2017). Along these lines, when middle-level managers perceive work-related benefits from top-level managers’ time-based leadership behaviors, they may learn how to implement such behaviors (Bandura, 1986) because they are worthy of imitation. As a result, we offer the following hypothesis:

*Hypothesis* 1: Top managers’ temporal leadership is positively related to middle-level managers’ temporal leadership.

### The mediating role of temporal leadership perspective

According to social learning theory, the learning process mediates the influences of the role models on followers (Bandura, 1977, 1986). That is, individuals (e.g., followers) develop a scheme on what kinds of attitudes, cognition, and behaviors from the role models (e.g., leaders) are valuable to learn (Bryan and Test, 1967; Rosenhan and White, 1967; Freeman and Martin, 2004; Mayer et al., 2009), and then they learn and imitate these attitudes, cognition, and behaviors (Bandura, 1986). In other words, it is simpler for individuals to recall evidence of behaviors associated with a role model and to anticipate what behaviors in similar circumstances are appropriate when they have a role model to look up to (Markus, 1977). Leadership research has suggested the trickle-down effects from managers to followers based on the logic of social learning theory (Bass et al., 1987; Mayer et al., 2009; Liu et al., 2012; Mawritz et al., 2012; Kwan, 2014).

To be clear, we first argue that top managers’ temporal leadership serves as a role model for middle-level managers, which is the foundation for the social learning process. Temporal leadership emphasizes a leader’s motivation and ability to manage temporal resources within an organization, and is particularly useful for helping organizations deal with significant environmental challenges (Shipp and Fried, 2014; Chen and Nadkarni, 2017). We suggest middle-level managers tend to view top managers’ temporal leadership as a role model to be followed given the benefit of timing in facilitating employees’ performance, team performance, and even organizational performance (Gevers et al., 2009; Op't Hoog, 2009; Mohammed et al., 2015; Mudannayake et al., 2016; Yuan and Lo, 2018).

Second, after establishing the modeling role of the leaders, followers then shape the schemas about reasonable beliefs, values, expectations, attitudes, and preferences (Rosenhan and White, 1967; Bandura, 1986; Paul et al., 2001; Wang and Rode, 2010). According to our theorizing, these appropriate attitudes, values, expectations, attitudes, and preferences toward temporal leadership can be reflected in temporal leadership perspective, which is defined as the willingness to construct, adjust, and manage the pace of task completion by the ministry. Under top managers’ temporal leadership, middle-level managers develop a set of beliefs and values that emphasize the leader’s role for managing the organization’s time resources. Like other kinds of schemas, the temporal leadership perspective produces a perceptual filter that influences how the information about the manager’s role is processed, categorized, and interpreted. It directs middle-level managers understanding what they should do as managers (Mohammed et al., 2015). For instance, middle-level managers might adopt a concept of being a time-leader manager, believing that providing time-resource-related support to employees is part of their job and can assist employees in completing their work tasks more efficiently.

Therefore, we propose that middle-level managers’ temporal leadership perspective mediates the relationship between the top managers’ temporal leadership and middle-level managers’ temporal leadership. Thus, we hypothesize,

*Hypothesis* 2: The positive relationship between top managers’ temporal leadership and middle-level managers' temporal leadership is mediated by middle-level managers' temporal leadership perspective.

### The moderating role of middle-level managers’ identification with the top manager

Although we anticipate that top managers’ temporal leadership will have a generally positive effect on middle-level managers’ temporal leadership perspectives, there is evidence that this relationship is dependent on some boundary conditions. For instance, the extent to which middle-level managers identify with their top managers as role models may influence how willing middle-level managers are to imitate the behaviors of their leaders.

Identification is primarily a construct of social identity. Initially, the social identity literature concentrated on explaining individual variations in psychological states (Albert and Whetten, 1985). Social identity is formed through categorizing individuals and encouraging behaviors that are congruent with the identity (Ashford et al., 1989). Furthermore, through schemas that direct their observation, expectation, and imitation of role-modeling behavior, identification motivates individuals to adopt their role model’s perspective on self and life (Pratt, 1998). In the supervisor-employee dyads, employees’ identification with their supervisor means having strong emotional attachments to their supervisor and incorporating the beliefs they share with their supervisor into their own identities. Thus, compared with those who have a low level of identification with the leader, followers who highly identify with their leaders are more likely to perceive the leader as a role model, consider what their leader demands of them, and utilize their leaders’ activities as a guideline for their future behavior (Pratt, 1998; Wang and Rode, 2010).

Similarly, social identity studies have shown that a leader who provides contextual cues to help employees make sense of their surroundings can shift individuals’ orientations away from personal interests and turn to the collective interests of others (Hogg and van Knippenberg, 2003). Middle-level managers who strongly identify with the top managers’ leadership behaviors may want to align with the top managers in the top manager-middle-level manager dichotomy. As a result, these middle-level managers are more susceptible to their top managers’ influence. They are thus more likely to perceive that their temporal leadership can also be endorsed by the top manager (Lord and Brown, 2004). By contrast, middle-level managers who do not identify with the top manager, on the other hand, are less likely to see their top manager as a role model and to internalize the top managers’ beliefs. This is consistent with social learning theory, which states that observational learning is governed by attentional processes (Bandura, 2004). That is, if a person does not pay attention to his or her role models’ behavior, he or she will not learn much from observation and will be much less likely to imitate their behavior (Bandura, 1977, 1986). As a result, we propose:

*Hypothesis* 3: Middle-level managers’ identification with the top manager moderates the relationship between top managers’ temporal leadership and middle-level managers’ temporal leadership perspective, such that the positive relationship is stronger when middle-level managers’ identification with the top manager is high rather than low.

### The moderated mediation model of identification with the top manager

Combining the preceding arguments yields a comprehensive framework in which middle-level managers’ identification with the top manager acts as a moderator, and the temporal leadership perspective mediates the positive relationship between the top managers’ temporal leadership and middle-level managers’ temporal leadership. We argue that using an interaction-based approach to role modeling effects (Wang and Rode, 2010), middle-level managers’ identification with the top manager will moderate the indirect effect of top managers’ temporal leadership on middle-level managers’ temporal leadership *via* middle-level managers’ temporal leadership perspective. By integrating the moderating effect of identification with the mediating effects of the temporal leadership perspective, we hypothesize that middle-level managers with higher levels of identification with the top manager have a greater degree of transmission of top managers’ temporal leadership than middle-level managers with lower levels of identification, and thus the effect of top managers’ temporal leadership on middle-level managers’ temporal leadership is likely to be stronger.

*Hypothesis* 4: The indirect effect of the top managers’ temporal leadership on middle-level managers’ temporal leadership via the temporal leadership perspective is moderated by identification with the top manager, such that the indirect effect is more positive when middle-level managers’ identification with the top manager is high rather than low.

## Materials and methods

### Sample and procedures

To test the proposed hypotheses, we contacted 99 firms located in China to collect multilevel, multiphase, and multi-source data. Before collecting the data, one of the authors scheduled face-to-face or telephonic meetings with the top managers and middle-level managers. At each meeting, the author outlined the purpose of our investigation, encouraged participation, and assured the firms’ middle-level managers and junior managers/employees that their responses would be confidential and that each participating firm would receive a summary when the study was finalized. The author also stated that we needed to hire administrative assistants in each firm to monitor the progress. We also asked the top managers to identify all of their middle-level managers, and middle-level managers to identify all of their junior managers/employees so that we could send a message to each of them to promote their involvement. Because the research has suggested that assistants (e.g., human resource assistants and administrative assistants) in a firm know the firm’s management and that such interpersonal connections can increase the response rate (Qian et al., 2013), we required human resource assistants or administrative assistants in each firm to help collect the data. One of the authors personally presented the surveys to these assistants, who then disseminated and collected responses from middle-level managers and junior managers/employees. We encouraged the middle-level managers and their junior managers/employees to send their surveys directly to our assistants in a manila envelope with no apparent identification to maintain confidentiality and privacy (such as name or title). We used phone calls and in-person visits to follow up with non-respondents.

From the 132 firms, 256 middle-level managers and 754 junior managers/employees agreed to participate in this study. The response rate was similar to a previous study (Chen and Nadkarni, 2017). To alleviate potential common method bias and strengthen the directional inferences of our model, we collected data in two waves, 2 weeks apart, and from two sources, namely, middle-level managers and junior managers/employees (Doty and Glick, 1998; Podsakoff et al., 2003). At Time 1, we delivered the surveys to the middle-level managers and collected their responses regarding the top managers’ temporal leadership, middle-level managers’ identification with the top manager, middle-level managers’ temporal leadership perspective, and demographics. We obtained responses from 106 firms. Two weeks later, at Time 2, we asked the junior managers/employees to complete surveys on middle-level managers’ temporal leadership. The middle-level managers identified the junior managers/employees and informed them of the surveys in advance. This process yielded valid responses from 99 firms, 234 middle-level managers, and 686 junior managers/employees. An average of 2.65 middle-level managers (59.69% of all potential middle-level managers) from each firm took part in the survey. An average of 3.01 junior managers/employees (67.03% of all potential junior managers/employees) from each firm participated in the survey.

The mean age of the 234 middle-level managers was 43.62 years (SD = 8.01), and 70 (70.0%) were men. The mean age of the 686 junior managers/employees was 27.56 years (SD = 6.14), and 377 (54.96%) were men. The number of employees at each firm ranged from 9 to 2,300. The average firm size was 149.54 persons (standard deviation = 338.32).

### Measures

To test our hypotheses, we used the following measures. To improve the validity of our questionnaires, we conducted a pilot test with 10 top managers in China (who were not in the sample) and modified the instructions based on their feedback. The questionnaires were back-translated from English to Chinese using standard procedures (Qian et al., 2013).

#### Temporal leadership

We employed Mohammed and Nadkarni (2011) seven-item scale. This scale offers good scale validity and has been applied to Chinese samples to test top managers’ temporal leadership (Chen and Nadkarni, 2017). Sample items include, “To what extent do the top managers of your firm pace the top management team so that work is finished on time?”; “To what extent are the top managers of your firm effective in coordinating the top management team to meet deadlines?”; and “To what extent do the top managers of your firm prepare and build in time for contingencies, problems, and emerging issues?” Each firm’s at least two middle-level managers scored the seven items on a 5-point scale (from 1 = not at all to 5 = a lot). We averaged the responses of the middle-level managers from each firm to obtain each top managers’ temporal leadership score. The Cronbach’s alpha for top managers’ temporal leadership was 0.85. The Cronbach’s alpha for middle-level managers’ temporal leadership was 0.91. We also measured middle-level managers’ temporal leadership (junior managers/employees rate middle-level managers’ temporal leadership), aggregated to team-level variables. For this purpose, we calculated the intra-group consistency Rwg(j), intra-group correlation ICC (1), and inter-group correlation ICC (2) for the two-time leaders separately to test the aggregation of variable data on whether cross-level studies can be conducted. Aggregation checks for the top managers’ temporal leadership scale yielded satisfactory results (Intraclass correlation coefficient ICC [1] =0.53; ICC [2] =0.73; mean rwg(j) = 0.91; *F* = 3.66, *p* < 0.001). While the mean Rwg(j) for middle-levels’ temporal leadership was 0.92, ICC [1] =0.79, and ICC [2] =0.89. According to Chan’s (1998) study of high-level variable measures, it can be seen that Rwg(j) > 0.7 and ICC (1) < ICC (2) both indicate that the consistency meets the acceptance criteria and the between-group differences are greater than the within-group differences, and both variables meet the data aggregation requirements and can be tested across strata.

#### Temporal leadership perspective

Refers to the willingness to construct, adjust, and manage the pace of task completion by the ministry. No previous research has been conducted on the temporal leadership perspective. Therefore, following Hinkin (1998) scale development procedure, this study carried out a pilot study to develop and validate a five-item scale of temporal leadership perspective. More specifically, we adopted a deductive multistage approach to developing a measure of temporal leadership perspective with a sample of 122 incumbent managers in companies. This measure was self-reported by respondents to mark their level of agreement with each statement. One example was, “As a supervisor, I believe I should be concerned about my subordinates to enable them to complete their work tasks within a specified time frame” and “As a supervisor, I believe I should be concerned about my subordinates to enable them to plan the sequence of completion of a series of work tasks “. We then used SPSS and Mplus software to conduct reliability, EFA, and CFA tests. Similar constructs to the temporal leadership perspective were distinguished: temporal leadership, controlling supervisory, mutual monitoring, time management, shared time cognitions, and temporal familiarity. The good fit indices indicated the scale’s convergent and discriminant validity. The Cronbach’s alpha for senior executives’ temporal leadership perspective was 0.93. We applied all five items of temporal leadership perspective in the main study. The measure was self-reported by senior executives and its Cronbach’s alpha was 0.89.

#### Identification with the supervisor

I adapted the 10-item scale of identification originally created by Kark et al. (2003) and utilized by Wang and Rode (2010) to measure identification with the supervisor by replacing “manager/supervisor” with “TOP MANAGER.” In this study, the measure was rated by middle-level managers. Identification with the top manager refers to the extent to which middle-level managers have a strong emotional commitment to them as well as incorporate their top managers’ values and views into their own identities. The 10 items highlight the identification characteristics, including “role model,” “similar values,” and “praise my top manager,” which are consistent with the conceptual definition. “When someone criticizes my top manager, it seems like a personal attack to me,” is an example item. For this measurement, Cronbach’s alpha was 0.94.

#### Control variables

We controlled for the top managers’ demographics (age, gender, education, and tenure; Wiersema and Bantel, 1992). We used the following dummy code values: 1 for males and 2 for females. Age was measured using four categories: 20 years or younger, 21 to 30 years, 31 to 40 years, and 41 to 50 years and older. The education level was measured using four categories: 1 for a high school education or less, 2 for a community college degree, 3 for a bachelor’s degree, 4 for a master’s degree, and 5 for a Ph.D. degree. We also controlled middle-level managers’ shared time cognitions, which in the study by (Santos et al., 2016) confirmed that leaders’ shared time cognitions have a positive effect on their temporal leadership. Thus, we also controlled for middle-level managers’ shared time cognitions in this study.

## Results

### Preliminary analysis

In this study, the Preacher et al. (2007) method was applied, and the multilevel structural equation modeling with Mplus 8.0 software was utilized to evaluate the mediated model that was being moderated. All outcome estimates were completed by Monte Carlo Simulation with more than 20,000 repeated random samples to obtain unbiased confidence intervals for the interaction terms (Preacher and Selig, 2012).

#### Descriptive statistics

The averages, standard deviations, and zero-order correlations for each of the key variables are shown in Table 1. Top managers’ temporal leadership was positively correlated with middle-level managers’ temporal leadership (*r = 0*.27*, p < 0*.01), and middle-level managers’ temporal leadership perspective (*r =* 0.32*，p < 0*.01). Middle-level managers’ temporal leadership perspective was positively correlated with middle-level managers’ temporal leadership (*r =* 0.16, *p < 0*.01). The relationships that had been hypothesized received some supportive evidence from these results.

**Table 1:** Means, standard deviations, and correlations.

**Variables**	**Mean**	**SD**	**1**	**2**	**3**	**4**	**5**	**6**	**7**	**8**
1.Middle-level managers’ age	38.56	6.87	1							
2. Middle-level managers’ tenure	13.69	7.74	0.82^**^							
3. Middle-level managers’ education	4.07	0.60	−0.06	−0.12^**^						
4. Middle-level managers’ gender	1.29	0.45	−0.21^*^	−0.22^**^	−0.17^**^					
5. Middle-level managers’ shared time cognitions	3.51	0.97	−0.17^*^	−0.12	0.04	0.02				
6. Middle-level managers’ identification with the top manager	3.41	0.87	−0.02	−0.05	−0.09^*^	0.00^*^	0.35^***^			
7. Middle-level managers’ temporal leadership perspective	3.55	0.86	−0.09^**^	−0.06	−0.10^**^	0.17^**^	0.17^**^	0.36^*^		
8. Middle-level managers’ temporal leadership	3.59	0.71	−0.07	−0.05	0.07	−0.04	0.27^**^	0.09^*^	0.32^**^	
9.Top managers’ temporal leadership	3.79	0.64	0.06	0.05	0.11^**^	−0.03	0.17^**^	0.18^**^	0.16^**^	0.27^**^

N middle-level managers = 234; N top managers = 99.

*** *p < 0.001;*

** *p < 0.01;*

* *p < 0.05, (two-tailed)*.

#### Confirmatory factor analysis (CFA)

**Figure 1:**
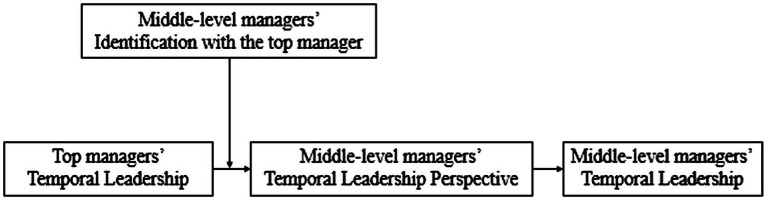
The conceptual model.

To evaluate the discriminant validity of the multi-item variables and the convergent validity of our measuring methodology (i.e., top managers’ temporal leadership, identification with the top manager, middle-level managers’ temporal leadership perspective, and middle-level managers’ temporal leadership), we used Mplus 8.0 to conduct a CFA (Muthén et al., 2017). As shown in Table 2, the CFA results indicated that the four-factor model had a good fit to the data (*χ2* = 106.42, *df* = 223; RMSEA = 0.03, CFI = 0.98, IFI = 0.98). Convergent validity is supported by the indicators’ substantial loading on their respective latent variables. In addition, we checked for discriminant validity by comparing the measurement model with four alternative models. Specifically, the standardized factor loadings for each topic in the four-factor model were significant at the 0.01 level, indicating good convergent validity for the construct measures, whereas the remaining four models (three-factor, two-factor, and one-factor models) fit the observed data poorly, and both model fit indices and chi-square tests revealed significant differences between the alternative and hypothetical models, with the hypothetical model having better convergent validity. The chi-square tests revealed that the four-factor model fit the data significantly better. The discriminant validity of the four multi-item variables was confirmed by these findings. The conceptual model of this paper is shown in Figure 1.

**Table 2:** Results of confirmatory factor analyses.

Model	χ^2^	*df*	RMSEA	CFI	TLI	Δχ^2^ (*df*)
**Four-factor model:** TMTL, IWTM, MMTP, MMTL	282.01	224	0.03	0.98	0.98	
**Three-factor model 1:** TMTL, IWTM, MMTLP+MMTL	589.58	227	0.08	0.86	0.85	307.57^**^ (3)
**Three-factor model 2:** TMTL+MMTLP, IWTM, MMTL	675.85	227	0.09	0.83	0.81	393.84^**^ (3)
**Two-factor model:** TMTL+MMTLP+MMTL, IWTM	1197.19	229	0.13	0.63	0.59	915.18^**^ (5)
**One-factor model:** TMTL+IWTM+MMTLP+MMTL	1616.70	230	0.16	0.47	0.41	1134.69^**^ (6)

TMTL = Top managers’ temporal leadership，IWTM = Identification with the top manager, MMTLP = Middle-level managers’ temporal leadership perspective，MMTL = Middle-level managers’ temporal leadership. **p < 0.01.

### Hypothesis testing

To evaluate our hypothesis, we implemented hierarchical regression analyses (Aiken et al., 1991). In Hypothesis 1, we predict that the top manager’s temporal leadership is positively and significantly associated with middle-level managers’ temporal leadership. Table 3 summarizes the findings. These results revealed that the top managers’ temporal leadership was positively and significantly associated with middle-level managers’ temporal leadership (*B* = 0.26, *SE* = 0.04, *p* < 0.001, Model 2) after we controlled the middle-level managers’ gender, age, educational level, and shared time cognition. Hypothesis 1 was confirmed.

**Table 3:** Results of hierarchical regression analysis.

Variables	Middle-level managers’ temporal leadership perspective				Middle-level managers’ temporal leadership				
	M1	M2	M3	M4	M5	M6	M7	M8	M9
	5.06	3.41	3.56	3.34	3.02	3.27	3.58	3.42	3.45
Middle-level managers’ age	0.01(0.01)	0.01(0.01)	0.00(0.01)	0.00(0.01)	0.00(0.01)	0.00(0.01)	0.00(0.01)	0.00(0.01)	−0.00(0.01)
Middle-level managers’ tenure	−0.02(0.01)	−0.02(0.01)	−0.01(0.01)	−0.01(0.01)	−0.02(0.01)	−0.02(0.01)	−0.02(0.01)	−0.02(0.01)	−0.01(0.01)
Middle-level managers’ education	−0.15(0.13)	−0.15(0.13)	−0.15(0.13)	−0.18(0.13)	−0.05(0.07)	−0.05(0.07)	−0.06(0.07)	−0.06(0.07)	−0.04(0.08)
Middle-level managers’ gender	−0.05(0.14)	−0.06(0.14)	−0.13(0.15)	−0.07(0.14)	−0.03(0.11)	−0.03(0.11)	−0.02(0.10)	−0.02(0.10)	−0.02(0.12)
Middle-level managers’ shared time cognition	0.13(0.07)	0.12(0.07)	0.04(0.07)	0.02(0.07)	0.17^**^(0.05)	0.17^**^(0.05)	0.18^**^(0.05)	0.18^**^(0.05)	0.15^*^(0.05)
Middle-level managers’ identification with the top manager			−0.02(0.03)	−0.10(0.03)			−0.02 (0.03)	−0.04(0.03)	−0.10^**^(0.03)
Middle-level managers’ temporal leadership perspective									0.19^***^(0.03)
Top managers’ temporal leadership		0.26^***^(0.04)	0.27^***^(0.04)	0.23^***^(0.04)		0.26^**^(0.04)	0.27^**^(0.04)	0.26^***^(0.04)	0.24^**^(0.04)
Top managers’ temporal leadership ×Middle-level managers’ identification with the top manager				0.25^***^(0.03)				0.20^***^(0.02)	0.16^***^(0.02)
*R2*	0.00	0.02	0.02	0.03	0.02	0.13	0.18	0.24	0.29
*ΔR2*		0.02	0.00	0.01		0.09	0.05	0.06	0.05
*F*	1.12	1.91	1.67	2.06	2.06	7.83	9.47	11.63	13.08

N middle-level managers = 234; N top managers = 99.

*** *p < 0.001;*

** *p < 0.01;*

* *p < 0.05, (two-tailed)*.

We utilized a t-test and bootstrapping with 20,000 replications to estimate the hypothesized indirect relationships. The results of the t-test and bootstrapping were highly consistent. According to the research process of Baron and Kenny (1986), the results of Model 2 from Table 3 indicate that there is a significant positive effect of top manager’s temporal leadership on middle-level managers’ temporal leadership perspective after controlling for the control variables (*B = 0*.26*, SE =* 0.04*, p <* 0.001). The results of model 9 indicate that middle-level managers’ temporal leadership perspective has a significant positive effect on middle-level managers’ temporal leadership (*B =* 0.19*, SE = 0*.03*, p <* 0.001). We utilized a t-test and bootstrapping with 20,000 replications to estimate the hypothesized indirect relationships. The results of the t-test and bootstrapping were highly consistent. According to the research process of Baron and Kenny (1986), the results of Model 2 from Table 3 indicate that there is a significant positive effect of top managers’ temporal leadership on middle-level managers’ temporal leadership perspective after controlling for the control variables (*B = 0*.26*, SE = 0*.04*, p < 0*.001). The results of model 9 indicate that middle-level managers’ temporal leadership perspective has a significant positive effect on middle-level managers’ temporal leadership (*B =* 0.19*, SE = 0*.03*, p <* 0.001).

**Figure 2:**
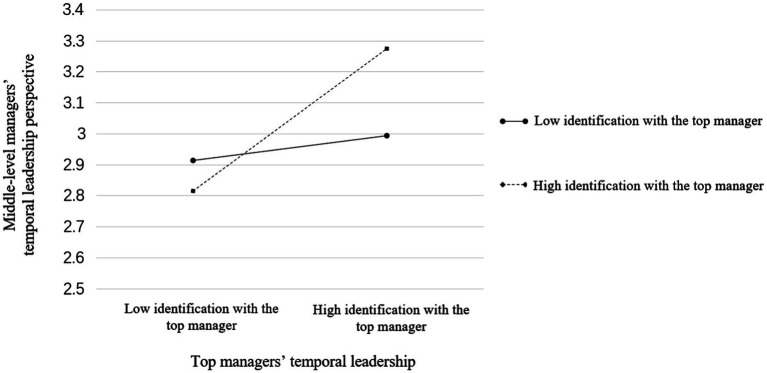
The moderating effect of middle-level managers’ identification with the top manager on the relationship between top managers’ temporal leadership and middle-level managers’ temporal leadership perspective.

According to Hypothesis 3, middle-level managers’ identification with the top manager moderates the relationship between the top managers’ temporal leadership and the temporal leadership perspective of middle-level managers. As demonstrated in Table 4, the interaction between top managers’ temporal leadership and middle-level managers’ identification with the top manager was correlated to middle-level managers’ temporal leadership perspective in a positive and significant way (*B = 0*.25*, SE = 0*.03*, p < 0*.001, Model 4). According to Aiken et al. (1991) suggestion, we further explained the mode of the moderating effect by plotting the relationship between the top managers’ temporal leadership and middle-level managers’ temporal leadership perspective at two levels of middle-level managers’ identification with the top manager, 1 standard deviation above or below the mean. In addition, the interaction between top managers’ temporal leadership and middle-level managers’ identification with the top manager was also positively and significantly related to middle-level managers’ temporal leadership (*B* = 0.16, *SE* = 0.02, *p* < 0.001, Model 9). As shown in Figure 2, top managers’ temporal leadership was more positively related to middle-level managers’ temporal leadership perspective for higher identification with the top manager (+1 *SD*; *B* = 0.29, *p* < 0.001) than for weaker identification with the top manager, and top managers’ temporal leadership was non-significate with middle-level managers’ temporal leadership perspective (−1 *SD*; *B* = −0.01, *n.s.*). The difference between the two levels was statistically significant (*∆B* = 0.08, *SE* = 0.04, *p* < 0.01). Hypothesis 3 was therefore strengthened.

**Table 4:** Results of the moderated path analysis.

Moderator	Top managers’ temporal leadership (X)→Middle-level managers’ temporal leadership perspective (M)→Middle-level managers’ temporal leadership (Y)		
	*B*	*SE.*	95% Unbiased confidence interval
Mean level: indirect effect	0.06	0.01	[0.04，0.10]
Simple paths for low identification of the top manager^a^ (-1*SD*)	−0.02	0.01	[−0.04，0.00]
Simple paths for high identification of the top manager^b^ (+1*SD*)	0.06	0.03	[0.03，0.09]
Differences^c^	0.08	0.04	[0.04，0.12]

N middle-level managers = 234; N top managers = 99.

a Low identification of the top manager refers to one standard deviation below the mean of identification of the top manager.

b High identification of the top manager refers to one standard deviation above the mean of identification of the top manager.

c Tests of differences for the indirect and total effect were based on bias-corrected confidence intervals derived from bootstrap estimates.

Hypotheses 4 propose the moderating effects of middle-level managers’ identification of the top manager on the relationships between top managers’ temporal leadership and middle-level managers’ temporal leadership through middle-level managers’ temporal leadership perspective. To test these relationships, we applied a bootstrap analysis and generated the CIs of the conditional indirect effect (Preacher et al., 2007). As shown in Table 4, the path from top managers’ temporal leadership to middle-level managers’ temporal leadership through middle-level managers was significant for high middle-level managers’ identification with the top manager (+1 *SD*; *B* = 0.06, *SE* = 0.03, 95% unbiased confidence interval of [0.03, 0.09], excluding 0), whereas the indirect effect was non-significant for low middle-level managers’ identification with the top manager (−1 *SD*; *B* = − 0.02, *SE* = 0.01, 95% unbiased confidence interval [−0.04, 0.00], including 0). The difference in indirect effects was statistically significant (*∆B = 0.08, SE = 0.04*, 95% unbiased confidence interval [0.04, 0.12], excluding 0). As a result, Hypothesis 4 acquired more support.

## Discussion

Employing Bandura's (1977, 1986) social learning theory, we revealed that the trickle-down impact of top managers’ temporal leadership on middle-level managers’ temporal leadership was exerted *via* middle-level managers’ temporal leadership perspective. Moreover, identification with the top manager strengthened the positive influence of top managers’ temporal leadership on middle-level managers’ temporal leadership perspectives, as well as the indirect effect of top managers’ temporal leadership on middle-level managers’ temporal leadership *via* middle-level managers’ temporal leadership perspectives. The findings of this study provide several significant theoretical contributions and management implications.

### Theoretical implications

Our findings add to the current research in four ways. First, we contribute to the literature on temporal leadership by exposing temporal leadership’s trickle-down effects. Although the study of temporal leadership has attracted increasing attention in recent years (Zhang et al., 2008; Mohammed and Nadkarni, 2011; Mohammed and Alipour, 2014; Maruping et al., 2015; Nadkarni et al., 2016; Puts, 2016; Santos et al., 2016; Chen and Nadkarni, 2017; Yuan and Lo, 2018), the mechanism of temporal leadership transmission at various levels in the organization has not been discussed by scholars. To the best of our knowledge, this is the first empirical study to look into why lower-level (e.g., middle-level) managers exhibit temporal leadership compared with upper-level managers (Chen and Nadkarni, 2017). By focusing on the temporal leadership of top managers, we analyzed the key aspects that can contribute to the occurrence of middle-level managers’ temporal leadership perspective to study predictors of middle-level managers’ temporal leadership. Furthermore, the importance of role modeling in the learning process has previously been identified by social learning theory (Bandura, 1986). We incorporate the role modeling process (i.e., role building, identification, and behavioral imitation) into examining the trickle-down effect of temporal leadership. By observing their supervisors, middle-level managers learn how to perform their leadership behaviors. They believe that learning their behavior from top managers can help them avoid trial and error behaviors (Lian et al., 2012).

Second, the antecedent conditions for temporal leadership have also received little attention (Chen and Nadkarni, 2017). Our study enriches the existing discussion on the antecedents of temporal leadership and can provide a more comprehensive picture for the research on the antecedents of temporal leadership. Our research not only explores the occurrence of temporal leadership behavior from leaders’ personal temporal traits, but also seeks the reasons for the formation of temporal leadership from external factors. We attempt to find traces from the discussion of schemas in social learning theory, and discuss that high-level temporal leadership behavior is a key factor in the formation of low-level managers’ temporal leadership behavior, in order to encourage scholars to investigate the antecedents of temporary leadership further.

Third, we propose and test the mediating role of the temporal leadership perspective using social learning theory, adding to the temporal leadership literature. Despite leadership research demonstrating that upper-level managers’ behaviors trickle down to middle-level managers and influence lower-level managers’ behaviors, the mediating mechanisms that directly validate the formation of this social learning process have not been investigated (e.g., Bass et al., 1987; Mayer et al., 2009). Specifically, since personal perspective encompasses memory, cognition, emotion, motivation, and action, this transfer of temporal perspective can be viewed as a social learning process. By integrating the concept of temporal leadership perspective into the temporal leadership trickle-down process, we respond to temporal leadership scholars’ call for more research on temporal cognition (e.g., Mohammed and Alipour, 2014; Santos et al., 2016). When middle-level managers receive temporal leadership from the top manager, they develop a leadership model that prioritizes the management, control, and allocation of subordinates’ time resources. As a result, they demonstrate temporal leadership behaviors to their subordinates. This aspect, which has not yet been covered in the vast body of research on time management and time efficiency, establishes a precedent for further study.

Finally, we extend the understanding of temporal leadership by revealing the boundary condition of trickle-down effects of temporal leadership. According to social learning theory, the identification process is an important factor in understanding the trickle-down effect of temporal leadership (Lord and Brown, 2004). The examination of an individual difference—middle managers’ identification with their leader—as a moderator of top managers’ temporal impact is a key contribution of this study. We combine social identity and social learning theories to develop a comprehensive model that shows how social identification might affect social learning’s course and outcomes by acting as a brake on its effects (*cf.* Mayer et al., 2009; Mawritz et al., 2012). As previously discussed, identification with the top manager is an intriguing moderator because a high level of identification represents a high degree of role modeling and should strengthen the impact of role modeling behavior. While the assumptions about the main effect and the mediating effect are consistent with social learning theory (Bandura, 1986), this theory also affirms that individuals who pay close attention to role models are more likely to interpret their behaviors favorably, internalize their values, and, as a result, imitate their behaviors. We find that temporal leader behaviors matter more when followers strongly identify with their supervisor.

### Practical implications

In practice, this study has significant implications for organizations and managers because more organizations and managers are concerned with managing time resources within the organization (Mohammed and Nadkarni, 2011; Mohammed and Harrison, 2013; Yuan and Lo, 2018), particularly as the world changes at an alarming rate. We inspired organizations by emphasizing the modeling roles of top managers’ temporal leadership. That is, if an organization wants to promote the idea of making full use of time resources throughout the organization, the behaviors of top management are critical. To be specific, we pointed out that middle-level managers will learn temporal leadership from top managers. Through the learning process, middle-level managers will view temporal leadership as a useful behavior for the company, and they will be more willing to engage in behaviors such as reducing organizational temporal conflict, assisting subordinates in assigning time pressure, setting schedules, organizing job assignments, and allocating time for unexpected events (Yuan and Lo, 2018), which will eventually contribute to job performance (Zhang et al., 2008), creativity (Chen and Nadkarni, 2017), and work engagement (Mudannayake et al., 2016). In this regard, we suggest that companies are supposed to offer training in this area to raise awareness of the value of temporal leadership. For instance, middle-level managers can participate in a training program in which companies’ top managers can conduct a speech about how to manage time resources. In addition, if managers who exhibit temporal leadership are rewarded and supported, the frequency of this effective leadership in organizations will increase, resulting in an environment that supports temporal leadership.

Another practical contribution is that we found that strengthening middle-level managers’ identification with the top manager is another useful method for promoting the diffusion of temporal leadership between the top manager and middle-level managers. The social identity theory (Pratt, 1998) argues that when subordinates identify with their leaders, they combine their self-concept with that of their leaders, resulting in the development of strong identification with their leader. According to our research, the positive relationship between top managers’ temporal leadership and middle-level managers’ temporal leadership becomes stronger when middle-level managers highly identify with the top leaders. Thus, when carrying out relevant training programs on temporal leadership, we suggest HR departments invite top managers who frequently display temporal leadership as the speakers, and those who highly identify with the speakers can participate in that program.

### Limitations and future research

Despite the powerful theoretical contributions and implications, this study has several limitations. First off, the design of this study precluded assessing the causality of several hypothesized relationships. Because this analysis did not account for baseline levels of the outcome variables, we cannot completely rule out the possibility of reverse causality, in which some observed relationships have the opposite causal direction from what was predicted. For example, middle-level managers who exhibit more temporal leadership may also have a more positive view of a top manager, believing that managers should engage in time-resource-supportive behaviors with their subordinates. They might notice more of this temporal leadership in their leaders because they might view the behavior of middle-level managers as an effective filter. As a result, middle-level managers may assess the top managers’ temporal leadership more highly, identify it earlier, and offer an explanation for its occurrence that is more implementation-relevant. Because these data are assessed by individuals from the same data source, this concern is especially relevant to the inverse relationship between top managers’ temporal leadership perspectives and middle-level managers’ temporal leadership perspectives.

These findings, however, identify several intriguing themes for future research. First, this study emphasizes the importance of role modeling in temporal leadership, which may help researchers investigate how and why leaders’ time-related behaviors influence followers’ time-related behaviors *via* social learning. Future research can build on this study’s model and use social learning theory to develop hypotheses about how followers’ time-related behaviors are influenced by leaders’ time-related behaviors *via* social learning mechanisms. For example, researchers could examine whether leaders’ time-related behaviors change followers’ schemes in terms of providing timely support. The findings could offer a theoretical foundation for organizations to train their employees in time-related behaviors.

Second, although the role modeling effect was tested at the top manager and middle-level managers’ binary level, this study is still uncertain as to whether there is a relationship between CEO temporal leadership and base managers’ temporal leadership. Additionally, we are unsure as to whether this kind of trickle-down effect can be generalized to the display of base managers’ temporal leadership. This means that the CEO’s temporal leadership may or may not filter down to the base managers’ temporal leadership, which could then have an effect on the firm as a whole. On the one hand, leadership research provides strong evidence that role modeling can occur at different binary levels (Bass et al., 1987; Mayer et al., 2009). Due to their unique leadership mindsets, middle-level managers frequently develop and implement policies and initiatives into implementation on their own.

Therefore, they may be influenced not only by top managers’ temporal leadership. Future research could investigate whether organizational climate and culture influence top managers’ temporal leadership imitation and transmission. In other words, top managers’ behaviors can shape the organizational climate or culture, influencing middle managers and other organizational members. According to recent leadership studies, ethical leadership by senior managers fosters an ethical climate that encourages employees to act in ways that advance the organization’s citizenship (Shin, 2012). As a result, we hypothesize that future research will focus on developing a temporal supportive climate or culture to improve managers’ temporal leadership. Future research could look into the relationship between temporal leadership and organizational climate or culture.

## Conclusion

By investigating the trickle-down effects of temporal leadership, we add to the literature on temporal leadership. We discovered that top managers’ temporal leadership activates middle-level managers’ temporal leadership through their temporal leadership perspective. The identification of middle-level managers with the top manager strengthens the relationship between top managers’ temporal leadership and the temporal leadership perspective of middle-level managers. Our findings highlight the trickle-down effects of temporal leadership, imploring both leaders and subordinates to recognize the significance of developing a high-quality temporal leadership relationship.

## Data availability statement

The raw data supporting the conclusions of this article will be made available by the authors, without undue reservation.

## Ethics statement

This study was reviewed and approved by College of Business, Shanghai University of Finance and Economics. All patients/participants were informed about study procedures by cover page of the questionnaire and provided their written informed consent to participate in this study.

## Author contributions

All authors listed have made a substantial, direct, and intellectual contribution to the work and approved it for publication.

## Conflict of interest

The authors declare that the research was conducted in the absence of any commercial or financial relationships that could be construed as a potential conflict of interest.

## Publisher’s note

All claims expressed in this article are solely those of the authors and do not necessarily represent those of their affiliated organizations, or those of the publisher, the editors and the reviewers. Any product that may be evaluated in this article, or claim that may be made by its manufacturer, is not guaranteed or endorsed by the publisher.
